# Ribosomal Protein S12 Hastens Nucleation of Co-Transcriptional Ribosome Assembly

**DOI:** 10.3390/biom13060951

**Published:** 2023-06-06

**Authors:** Margaret L. Rodgers, Yunsheng Sun, Sarah A. Woodson

**Affiliations:** 1 Thomas C. Jenkins Department of Biophysics, Johns Hopkins University, Baltimore, MD 21218, USA; 2 The Laboratory of Biochemistry and Genetics, The National Institute of Diabetes and Digestive and Kidney Diseases, The National Institutes of Health, Bethesda, MD 20892, USA

**Keywords:** ribosome assembly, RNA chaperones, single-molecule fluorescence, ribosomal protein S12, co-transcriptional RNA folding

## Abstract

Ribosomal subunits begin assembly during transcription of the ribosomal RNA (rRNA), when the rRNA begins to fold and associate with ribosomal proteins (RPs). In bacteria, the first steps of ribosome assembly depend upon recognition of the properly folded rRNA by primary assembly proteins such as S4, which nucleates assembly of the 16S 5′ domain. Recent evidence, however, suggests that initial recognition by S4 is delayed due to variable folding of the rRNA during transcription. Here, using single-molecule colocalization co-transcriptional assembly (smCoCoA), we show that the late-binding RP S12 specifically promotes the association of S4 with the pre-16S rRNA during transcription, thereby accelerating nucleation of 30S ribosome assembly. Order of addition experiments suggest that S12 helps chaperone the rRNA during transcription, particularly near the S4 binding site. S12 interacts transiently with the rRNA during transcription and, consequently, a high concentration is required for its chaperone activity. These results support a model in which late-binding RPs moonlight as RNA chaperones during transcription in order to facilitate rapid assembly.

## 1. Introduction

**Figure 1:**
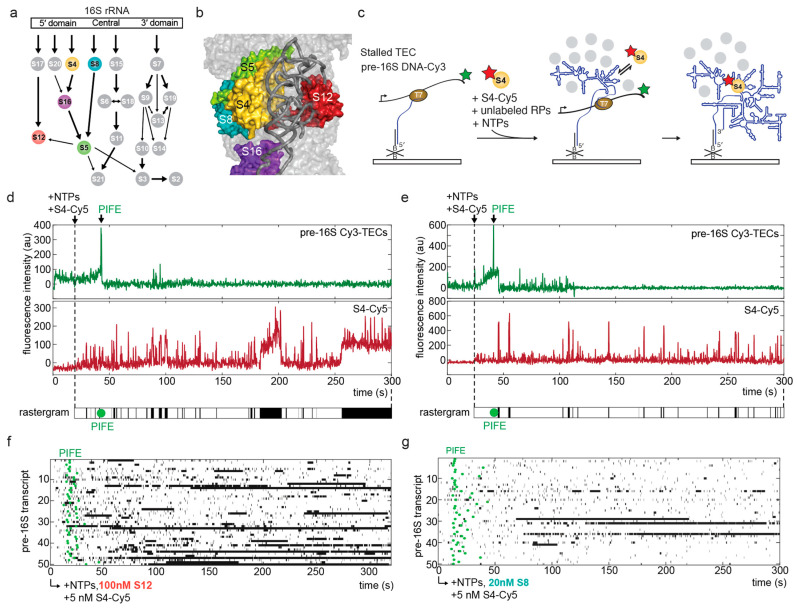
Single-molecule system to study the association of S4 in the presence of other RPs. (**a**) Nomura 30S ribosome assembly map highlighting RPs used in this study. (**b**) RPs surrounding the S4 binding site in the mature 30S ribosome. The 5WJ recognized by S4 is shown as a dark grey ribbon. PDB: 4V4A. (**c**) Schematic of the single-molecule system used to study the binding dynamics of S4 during and after transcription of the pre-16S rRNA. Green star, Cy3; red star, Cy5. (**d**,**e**) Sample raw single-molecule traces illustrating PIFE at the end of transcription (green; top) and colocalization of S4-Cy5 with the transcript (red; bottom). A rastergram for each transcript is shown below each plot of Cy5 intensity. This simplified annotation is used to visualize the timing of S4-Cy5 binding (black bars). PIFE (green circle) indicates the end of transcription. See Methods for details. (**f**,**g**) Rastergrams for 50 randomly selected pre-16S transcripts during and after transcription in the presence of (**f**) 100 nM unlabeled S12 and (**g**) 20 nM unlabeled S8. See Figure S2 for data with other RPs.

In all kingdoms of life, the structures of ribosomal subunits derive from the three-dimensional organization of the rRNA in complex with more than 20 unique ribosomal proteins (RPs) [1]. The RPs not only stabilize the rRNA in its native conformation, but also induce conformational changes in the rRNA that favor the next steps of assembly [2,3,4,5]. This linkage between rRNA folding and RP binding produces a hierarchy of protein addition that ensures the cooperativity of assembly (Figure 1a) [6,7]. In this hierarchy, primary assembly proteins associate with the naked rRNA, whereas secondary and tertiary assembly proteins only join the complex after a primary assembly protein has bound. This hierarchy is not strict, however, as 30S and 50S assembly can proceed via alternative paths [3,8,9].

In cells, RPs begin to associate with the pre-rRNA as it is being transcribed [10]. Footprinting experiments on refolded rRNA showed that the path of RP assembly at 30 °C in vitro is aligned with the 5′ to 3′ direction of transcription [11], although assembly can begin in any domain [4]. During transcription, assembly is likely nucleated by primary assembly RPs uS17, uS20, and uS4 that bind the 16S 5′ domain, since this domain is transcribed first. Protein uS4 (S4 hereafter) binds a five-way helix junction (5WJ) in the 16S rRNA, stabilizes RNA tertiary interactions throughout the 16S 5′ domain [12], and nucleates further assembly of the 5′ and central domains [7,13,14,15]. After S4 binds the 5WJ, it induces a conformational change in 16S h18 [16,17], an element of the 30S decoding site that is also stabilized by RP uS12 (S12) (Figure S1).

We previously used single-molecule fluorescence co-localization co-transcriptional assembly (smCoCoA) to visualize association of RP S4 with the rRNA during transcription [18]. Although native S4-rRNA complexes are stable at 20 mM MgCl_2_ (t^1/2^ >> 10 min) [5,13,19], most S4 binding events during transcription are unstable (<2 s) due to variable folding of the rRNA during transcription [18,20]. Interestingly, the likelihood of stable S4 binding during transcription increased when other RPs, including uS5 (S5), uS8 (S8), S12, bS16 (S16), uS17 (S17), and bS20 (S20), were also present.

These observations suggested that ribosomal proteins can accelerate rRNA folding and assembly in cells. Yet, the mechanism of this chaperone effect remains unknown. S17 and S20 bind to different helix junctions in the 16S 5′ domain, whereas S5, S8, S12, and S16 bind near S4 in the mature 30S subunit (Figure 1a,b). Therefore, these RPs may facilitate S4 binding by altering the conformational landscape of the 5′ domain. Additionally, RPs and other RNA chaperones could restructure the S4 binding site by unfolding an incorrect secondary structure, or by transiently stabilizing the tertiary structure of helix junctions [21].

Here, we use our co-transcriptional assembly assay to understand how additional RPs accelerate proper recognition of the S4 binding site, which is a key first step in 30S ribosome assembly. The results show that the conserved late-binding protein S12 promotes S4 association during transcription by acting on the nascent rRNA. S12 was previously found to have RNA chaperone activity, and may help the rRNA refold [22]. Interestingly, S12 binds the same rRNA 5WJ as S4, but on the opposite side. Thus, certain RPs may perform the dual functions of stabilizing native rRNA structure and accelerating rRNA refolding.

## 2. Materials and Methods

### 2.1. Preparation of Fluorescent DNA Templates

Pre-16S DNA template for transcription was prepared by PCR using a reverse primer containing a fluorescently labeled nucleotide, as previously described [18]. Fluorescent labeling was carried out by reacting an internal C6-amino-modified T (IDT, Coralville, IA, USA) with Cy3-NHS mono reactive dye (Lumiprobe Corp., Hunt Valley, MD, USA), as follows: 1 mg Cy3-NHS mono reactive dye was dissolved in 33 µL DMSO and added to a reaction mixture containing 10 nmol oligonucleotide, adjusted to 100 µL final volume with 100 mM sodium bicarbonate pH 8.5. Labeling reactions were incubated at room temperature for 24 h. Reactions were purified using a Nucleospin column (Takara Bio, San Jose, CA, USA). Fluorescent DNA transcription templates were generated by PCR using Q5 high-fidelity polymerase (NEB, Ipswich, MA, USA). Following PCR, fluorescently labeled DNA templates were separated on 1% agarose, and isolated from the gel using a Nucleospin gel purification kit (Takara Bio).

### 2.2. Protein Purification and Fluorescent Labeling

Unlabeled ribosomal proteins uS8 (S8), uS5 (S5), S12, and bS16 (S16) were expressed and purified as previously described [23,24]. T7 RNA polymerase (RNAP) was recombinantly expressed in *E. coli* BL21 (DE3) cells as previously described [25], and natively purified on a P11 phosphocellulose column followed by a Blue Dextran-Sepharose column, using a protocol developed for SP6 polymerase [26].

Fluorescently labeled S4:C32S,S189C was purified and labeled as previously described [5]. Briefly, S4:C32S,S189C was incubated for 3 h with a six-fold molar excess of dye in 80 mM K-HEPES pH 7.6, 1 M KCl, 1 mM TCEP, and 3 M urea at 20 °C. Unreacted dye was removed by cation exchange followed by dialysis against 80 mM K-HEPES pH 7.6, 1 M KCl, and 6 mM 2-mercaptoethanol (BME).

The S12:A48C expression plasmid was a kind gift from the Noller lab. Fluorescent S12:A48C was prepared and fluorescently labeled as previously described, but with a few modifications [27]. Briefly, S12:A48C was expressed in BL21 (DE3) cells. Upon lysis, S12 was found primarily in inclusion bodies. The inclusion bodies were dissolved in 10 mL buffer A (6 M guanidinium-HCl, 1 M KCl, 6 mM BME), cleared by centrifugation, and the soluble protein was dialyzed against 1 L buffer B (20 mM Na-acetate pH = 5.6, 1 M KCl, 6 M urea, 6 mM BME), 1 L buffer C (20 mM Na-acetate pH = 5.6, 500 mM KCl, 6 M urea, 6 mM BME), and twice against 1 L buffer D (20 mM Na-acetate pH = 5.6, 100 mM KCl, 6 M urea, 6 mM BME), for 2 h at each buffer change. The dialyzed protein was cleared by centrifugation and filtered (0.45 μm), then applied to a UNO-S6 column (Bio-Rad, Hercules, CA, USA) and eluted with a 0–40% linear gradient of 1 M KCl in buffer D. Half of the protein was dialyzed against buffer 1 (80 mM HEPES, pH = 7.6, 20 mM MgCl_2_, 1 M KCl, 6 mM BME) for 2 h, twice, flash frozen in aliquots and stored at −80 °C. The other half was dialyzed overnight against buffer 2 (80 mM HEPES pH = 7.5, 1 M KCl, 1 mM TCEP) for labeling.

For labeling with Cy5, S12 protein was warmed to 20 °C and diluted with buffer 1 to 1.7 mL total (40 μM S12 final). Cy5-maleimide mono-reactive dye (GE-Healthcare, Chicago, IL, USA) was dissolved in DMSO to 20 mM and immediately added to the protein solution. The reaction was incubated at 20 °C in the dark for 2 h. BME was added to 0.5% *v*/*v* to quench the reaction. The labeled protein was purified on an UNO-S6 column with 0–60% linear gradient of 1 M KCl in 10 mM Tris-HCl pH = 6.3, 6 M urea, 0.01% Nikkol, and 6 mM BME, with elution at 460 mM KCl. The protein was dialyzed against buffer 1 and stored at −80 °C as above.

### 2.3. Single-Molecule Fluorescence Microscopy

Single-molecule smCoCoA experiments were carried out as previously described [18]. Briefly, single-molecule microscopy was performed on a custom-built prism-based total internal reflection fluorescence microscope. Cy3-labeled biomolecules were imaged using a green (532 nm) laser and Cy5-labeled biomolecules were imaged using a red (640 nm) laser.

Stalled transcription elongation complexes (TECs) were assembled at RT for 2 min in the following reaction: 50–100 nM Cy3-labeled DNA template, 40 mM Tris-HCl pH 7.5, 20 mM MgCl_2_, 50 nM T7 RNAP, 200 µM GTP, 200 µM ATP, 50 µM UTP, 2 U RNasin Plus, and 100 nM biotinylated tether oligomer. Stalled TECs (20 µL) were diluted 1:10 in transcription buffer (40 mM Tris-HCl pH 7.5, 20 mM MgCl_2_), immobilized on quartz slides passivated with DDS-Tween20 [28], and functionalized with streptavidin. Following immobilization, stalled TECs were washed with imaging buffer (40 mM Tris-HCl pH 7.5, 20 mM MgCl_2_, 150 mM KCl, 1% *w*/*v* glucose, 165 U/mL glucose oxidase, 4 mM Trolox, 2 U RNasin Plus).

Before imaging, the restart imaging solution was assembled as follows: 40 mM Tris-HCl pH 7.5, 20 mM MgCl_2_, 150 mM KCl, 5 nM Cy5-S4 or Cy5-S12, 1 mM ATP, 1 mM GTP, 1 mM CTP, 1 mM UTP, 1% *w*/*v* glucose, 165 U/mL glucose oxidase, 2170 U/mL catalase, 4 mM Trolox, and 2 U RNasin Plus, with additional RPs as stated. The restart imaging solution was injected to the slide chamber during imaging. Imaging was performed with alternating frames of green and red excitation every 100 ms for a total of ~3000 frames (5 min).

### 2.4. Analysis of Single-Molecule Data

Single-molecule movies were analyzed as previously described [18] using Imscroll software [29]. Briefly, Cy3-labeled TECs were selected as areas of interest (AOIs) at the beginning of the movie. Colocalized spots were determined by translating the Cy3-TEC AOI locations to the Cy5 channel using a mapping function, as previously described [29]. The intensities for AOIs in both channels were integrated over the duration of the movie to generate single-molecule time traces. PIFE and Cy5-S4 colocalization was not observed in control reactions without NTPs, confirming that these signals report on transcription elongation and Cy5-S4 binding to the transcript, respectively [18].

Single-molecule traces were examined for transcription of the pre-16S rRNA as indicated by protein-induced fluorescence enhancement (PIFE), as previously described [18]. Only traces exhibiting a single PIFE signal were included in the analysis of colocalized Cy5-labeled RP to ensure that the analysis was limited to single, full-length pre-16S transcripts. Binding intervals for Cy5-labeled RPs were generated as previously described [29]. Dwell times lasting for a single frame (0.2 s) were indistinguishable from nonspecific binding of S4 or S12 with the slide surface in the absence of the RNA and were not included in the analysis. Maximum likelihood estimation (MLE) was used to globally fit the unbinned kinetic data using single- and triple-exponential kinetic binding models, as in Equations (1) and (2), respectively, where *x* is the duration of the binding event; *t*_m_ is the minimum resolvable time interval in the experiment; *t*_x_ is the maximum time interval; *τ*, *τ*_1_, *τ*_2_, *τ*_3_ represent characteristic lifetimes; and *a*_1_ and *a*_2_ are the amplitudes associated with the fitted lifetimes.

(1) $$\frac{1}{\left(e^{-\frac{t_{m}}{\tau }}-e^{-\frac{t_{x}}{\tau }}\right)}\cdot \frac{1}{\tau }e^{-\frac{x}{\tau }}$$

(2) $$\frac{1}{\begin{array}{c} a_{1}\left(e^{-\frac{t_{m}}{\tau_{1}}}-e^{-\frac{t_{x}}{\tau_{1}}}\right)+a_{2}\left(e^{-\frac{t_{m}}{\tau_{2}}}-e^{-\frac{t_{x}}{\tau_{2}}}\right)+\left(1-a_{1}-a_{2}\right)\left(e^{-\frac{t_{m}}{\tau_{3}}}-e^{-\frac{t_{x}}{\tau_{3}}}\right)\\ \cdot \left(\frac{a_{1}}{\tau_{1}}e^{-\frac{x}{\tau_{1}}}+\frac{a_{2}}{\tau_{2}}e^{-\frac{x}{\tau_{2}}}+\left(1-a_{1}-a_{2}\right)e^{-\frac{x}{\tau_{3}}}\right) \end{array}}$$

Errors in MLE parameters were estimated by bootstrapping the data to obtain a 95% confidence interval, as previously described [29]. Histograms were generated in MATLAB (the Mathworks) by unequal binning of the data to minimize empty bins and visualize the MLE fits. Error bars in the histogram represent the variance in a binomial distribution, described by Equation (3), where *N* is the number of observations and *P* is the event probability.

(3) $$\sigma =\sqrt{NP(1-P)}$$

Cumulative density plots were generated in MATLAB and single-exponential fitting of association times was carried out using GraphPad Prism.

## 3. Results

### 3.1. A Single-Molecule System to Study the Chaperone Activity of Ribosomal Proteins

Previous single-molecule experiments showed that the presence of secondary and tertiary RPs increase the likelihood that protein S4 binds the 16S rRNA stably during transcription (Figure 1a,b; [18]). This effect was greatest for a combination of proteins that bind the 16S 5′ domain (S4, S12, S16, S17, S20) and the adjacent central domain (S5, S8). By contrast, protein uS9 (S9) that binds the 16S 3′ domain had no effect on the likelihood of stable S4 binding.

In order to understand how each RP acts on the rRNA, we used single-molecule colocalization assembly (smCoCoA) to simultaneously monitor transcription of the pre-rRNA and association of Cy5-labeled S4 with the nascent RNA (Figure 1c). We used the stability of S4-Cy5 binding events as a readout for proper folding of the pre-16S rRNA, and measured how S4 binding dynamics changed in the presence of individual RPs.

To observe synthesis of the pre-rRNA in real time, we first assembled stalled T7 RNA polymerase transcription elongation complexes (TECs) on a Cy3-labeled pre-16S DNA template. Stalled TECs were immobilized on a microscope slide using a biotinylated DNA oligonucleotide that was complementary to the 5′ end of the nascent RNA (Figure 1c). Transcription was restarted during imaging by adding NTPs together with S4-Cy5 and unlabeled RPs in order to monitor S4-Cy5 binding dynamics during elongation of the pre-rRNA (Figure 1c). The end of transcription was marked by protein-induced fluorescence enhancement (PIFE), which occurs as RNAP passes over the Cy3 fluorophore attached to the DNA template, as described previously [18].

We first examined whether RPs that bind near S4 in the mature ribosome contribute to S4-Cy5 recruitment (Figure 1a,b). S5, S8, S12, or S16 was added to the transcription reaction at a concentration that mimics 30S reconstitution conditions: Primary protein S8 was added at 20 nM, while tertiary proteins S5 and S12 were added at 100 nM. Most S4 binding events were brief (Figure 1d,e), consistent with previous findings that improper pre-rRNA folding during transcription results in unstable or non-specific S4 binding [18]. Interestingly, we observed that the presence of S12 increased the number of stable S4 binding events compared to S4 alone, or to S4 with other RPs, such as S8 (Figure 1f,g and Figure S2).

### 3.2. S12 Increases Likelihood of Stable S4 Recruitment to Pre-16S rRNA during Transcription

**Figure 2:**
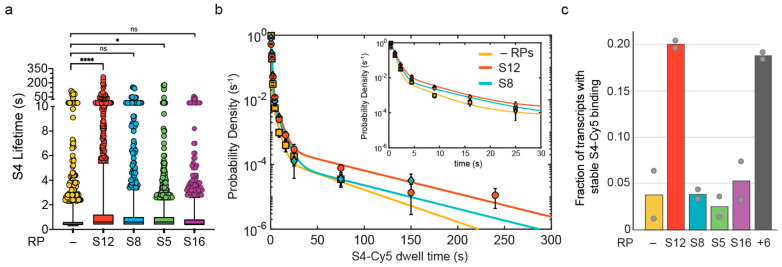
Stable binding of S4 is enhanced by S12. (**a**) Box plot of the distribution of S4-Cy5 dwell times in the presence of no other protein (–), 100 nM S12, 20 nM S8, 100 nM S5, and 50 nM S16. (****, *p* ≤ 0.0001; *, *p* ≤ 0.05; ns, *p* > 0.05; Student’s *t*-test) (**b**) Maximum likelihood analysis of the unbinned dwell time distribution shown in (**a**). Triple-exponential fit is shown with a colored line, and fitting parameters are reported in Table 1. Centers of the binned dwell time data for S12, S8, and –RPs are shown as circles, diamonds, and squares, respectively. See Figure S3 for additional data. (**c**) Fraction of pre-16S TECs that experienced an S4 binding event lasting longer than 20 s, for experiments as in (**a**). For comparison, the grey bar (+6) indicates the effect of adding six RPs at the same time: 20 nM S20, 20 nM S17, 20 nM S8, 50 nM S16, 100 nM S5, 100 nM S12; data from Ref. [18]. Bars, average; grey circles, individual replicates. See Table 1 for the number of transcripts analyzed in each experiment.

In order to characterize the contribution of each RP on the binding dynamics of S4-Cy5, we measured the distribution of S4-Cy5 dwell times, which report on the stability of S4-rRNA complexes in the presence of another RP. Because the S4-Cy5 dwell time distribution is dominated by short events, the mean dwell time ~1 s was similar for S4 alone and S4 in the presence of S5, S8, or S16. However, the mean was slightly higher in the presence of 100 nM S12 (~2 s), and the longest dwell times were more represented with 100 nM S12 compared to S4 alone (Figure 2a).

Because S4 interacts much more tightly with the properly folded 5WJ than with unstructured RNA [15,18,19], an increase in stable S4-Cy5 binding indicates that the rRNA is becoming better folded, on average. Therefore, the proportion of stable S4-Cy5 complexes can be used as a readout for changes in the folding quality of the rRNA. We used maximum likelihood analysis of the dwell time distributions to determine the characteristic dwell times associated with S4-Cy5 binding in the presence of each RP, and their amplitudes (Figure 2b and Figure S2). Consistent with previous work [18], we found that S4-Cy5 exhibits dwell times of $\tau_{1}$~0.5 s, $\tau_{2}$~6 s, and $\tau_{3}$ > 20 s (Table 1), which represent the inherent lifetimes of the different S4 binding modes. When only S4-Cy5 was added to the transcription reaction, the amplitude of the most stable binding mode, $a_{3}$, was very small (0.3%; Table 1). When S12 was also present, however, $a_{3}$ was sixfold higher, indicating that S4-Cy5 binding was six times more likely to produce a stable complex.

We next examined the fraction of pre-16S transcripts that formed a long-lived S4-Cy5 complex (>20 s) at some point during each movie (Figure 2c). This fraction reports on the probability that the pre-16S rRNA is competent for native-like S4 binding, which can be reliably estimated from the single transcripts. The results showed that 100 nM S12 increased the fraction of competent pre-16S transcripts by fourfold compared to other RPs and S4 alone (red bar, Figure 2c), suggesting that S12 generates a more native-like binding site for S4.

### 3.3. S12 Only Interacts Transiently with Pre-16S in the Absence of Other Ribosomal Proteins

We considered that S12 must bind the rRNA to facilitate folding of the 5WJ and stable binding of S4. One explanation for this effect is that S12 binds the rRNA together with S4 to facilitate folding of the 5WJ (co-binding mechanism). Another explanation is that S12 transiently binds the rRNA and changes its folding path, leaving the rRNA in a more native-like structure that is competent to stably bind S4 (independent binding mechanism). To distinguish between these mechanisms, we sought to measure how S12 binds the rRNA on its own.

**Figure 3:**
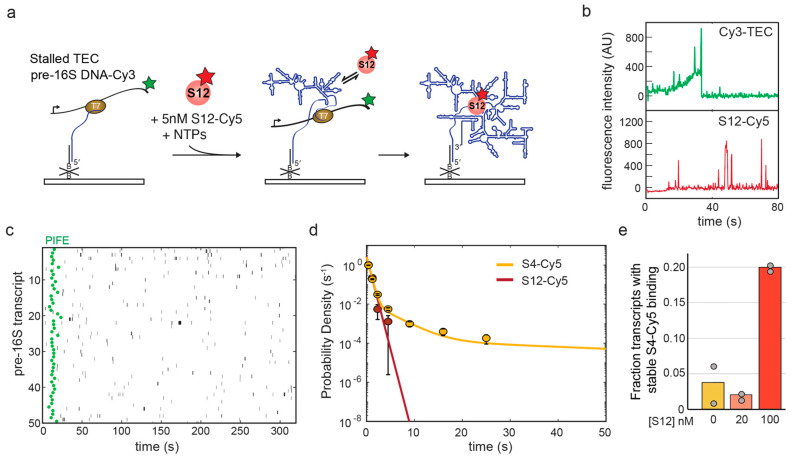
S12-Cy5 only interacts transiently with pre-16S transcripts in the absence of other RPs. (**a**) Single-molecule assay to measure S12 binding during transcription. (**b**) Example of a single-molecule trace illustrating transcription from a single Cy3-TEC (green) and colocalization of S12-Cy5 (red). (**c**) Rastergram of S12-Cy5 binding (black bars) to 50 randomly selected pre-16S TECs, as in Figure 1. (**d**) MLE analysis of S12-Cy5 binding to pre-16S transcripts (red) compared to S4-Cy5 (gold). The characteristic S12-Cy5 dwell time is τ = 0.46 s; see Table 1 for S4-Cy5 parameters. (**e**) The fraction of pre-16S TECs with stable S4-Cy5 binding is dependent on S12 concentration, as 20 nM unlabeled S12 does not improve S4-Cy5 binding.

In order to characterize the binding dynamics of S12 directly, we labeled S12 with a Cy5 fluorophore and carried out smCoCoA experiments on pre-16S TECs in the same manner as for S4-Cy5 (Figure 3a). We expected that S12 alone should not interact stably with the rRNA, because addition of S12 to the 16S rRNA requires many other RPs, including S4, in the Nomura assembly map. Consistent with the Nomura map, the single-molecule results showed that S12 only binds pre-16S rRNAs transiently under the conditions tested (Figure 3b,c). We measured the dwell times of S12-Cy5 complexes and found that the characteristic dwell time was τ~0.5 s, similar to non-specific S4-Cy5 binding to pre-16S rRNA (Figure 3d).

Since S12-Cy5 binds the rRNA only for a short time, we reasoned that the effect of S12 on S4-Cy5 binding may be concentration dependent for either co-binding or independent binding mechanisms. In order to examine the concentration dependence, we measured S4-Cy5 binding dynamics in the presence of 20 nM S12, and found that this amount of S12 was insufficient to improve S4 binding (Figure 3e). These results indicated that S12 must be present at a high concentration to facilitate the formation of stable S4-rRNA complexes through frequent, low-affinity interactions with the rRNA.

### 3.4. S12 Can Act on Pre-16S during and after Transcription to Increase Stable S4 Recruitment

In all aforementioned experiments, S12 was allowed to access the rRNA during and after transcription. Therefore, it was unclear if S12 helps the rRNA fold as it is synthesized or if S12 helps the rRNA refold after it has already misfolded.

**Figure 4:**
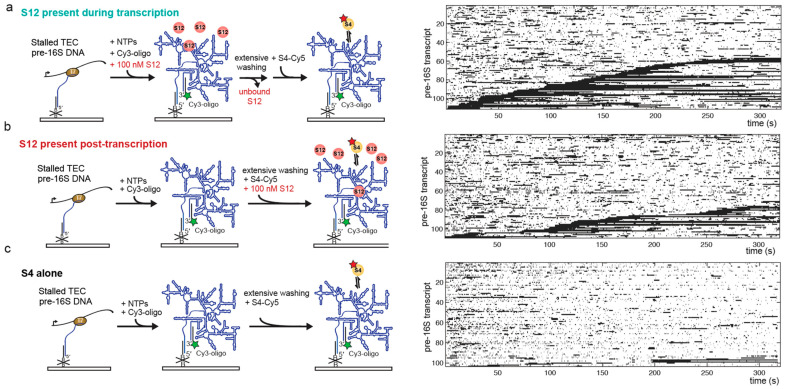
S12 is more effective when present during transcription. Strategy for visualizing S4-Cy5 binding to the pre-16S after transcription. Immobilized transcripts were detected by hybridization of a complementary Cy3-oligomer. (**a**) Binding to pre-16S transcribed in the presence of 100 nM S12. Rastergram of S4-Cy5 binding at right. Pre-16S transcripts were ordered by the start of the first S4-Cy5 binding event longer than 20 s. (**b**) Binding in the presence of 100 nM S12, as in (**a**). (**c**) Binding in the absence of S12, as in (**a**). See Figure S4 and Table S1 for binding lifetimes.

In order to examine whether S12 acts during or after transcription, we varied the order in which S12 is added to the pre-rRNA in the single-molecule experiments. First, we immobilized unlabeled stalled pre-16S TECs and injected NTPs to restart transcription, as described above (Figure 4). For co-transcriptional S12 interactions (Figure 4a), we added 100 nM S12 together with the NTPs to allow S12 to help fold the rRNA during transcription.

Following transcription, the slide was thoroughly washed to remove unbound S12 and NTPs from the reaction chamber. The co-transcriptionally folded pre-16S rRNAs were then labeled in situ with a Cy3-labeled oligomer that is complementary to the 3′ end of the pre-16S rRNA. Binding of S4-Cy5 to S12-treated transcripts was then monitored following injection of S4-Cy5 into the slide chamber during imaging (Figure 4a). To determine whether S12 can act after transcription, 100 nM S12 was added together with S4-Cy5 to previously transcribed pre-16S pre-rRNA (Figure 4b). Finally, we compared the results to a control in which S4-Cy5 was allowed to bind the pre-rRNA post-transcription in the absence of S12 (Figure 4c). We observed that adding S12 during transcription or after transcription increased the amount of stable S4-Cy5 binding compared to S4 alone (Figure 4). Moreover, S12 was able to enhance stable S4-Cy5 binding even after S12 was washed out (Figure 4a), supporting the idea that S12 acts on the pre-rRNA rather than on S4.

**Figure 5:**
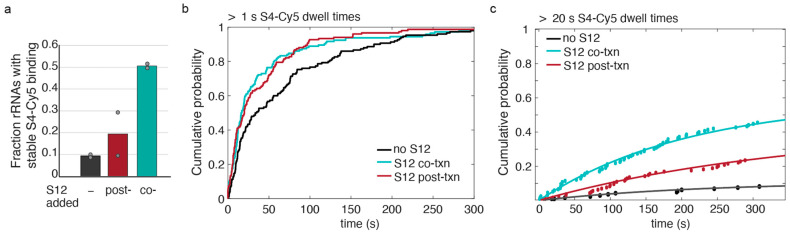
The presence of S12 influences S4 binding during transcription and after transcription. (**a**) Fraction of pre-16S rRNAs that bind S4 > 20 s in the absence of S12 (black bar; as in Figure 4c), in the presence of S12 after transcription (red bar; as in Figure 4b), and in the presence of S12 during transcription (teal bar; as in Figure 4a). Gray symbols indicate the values for independent replicates. (**b**) Cumulative probability plot of S4-Cy5 arrival times for specific events lasting >1 s. Association times were combined from two independent replicates. Association of S4-Cy5 is faster in the presence of S12 added during or after transcription. The cumulative density functions for S12 co-txn and S12 post-txn are statistically similar, and both are statistically different than no S12 (K–S test). (**c**) Cumulative probability plot of S4-Cy5 arrival times for stable events lasting >20 s. Apparent association times were fit with a single exponential function (lines). Stable association of S4-Cy5 is enhanced by the presence of S12 during and after transcription.

In order to determine whether S12 was more effective during or after transcription, we compared the fraction of pre-16S rRNAs competent to stably bind S4-Cy5 when S12 was added co-transcriptionally (co-txn) or post-transcriptionally (post-txn; Figure 5). The fraction of competent pre-16S rRNA increased nearly fivefold when S12 was added during transcription, compared with ~twofold when S12 was added post-transcription (Figure 5a). These data suggested that S12 more efficiently chaperones rRNA folding as it is synthesized compared to when the RNA has had time to misfold.

Next, we examined the kinetics of S4-Cy5 association with the rRNA when S12 was added, either during transcription (co-txn) or simultaneously with S4-Cy5 after transcription (post-txn). Previous experiments established that S4-Cy5 dwell times longer than 1 s mainly represent specific interactions with the 5WJ in the 16S rRNA [18].

Therefore, we first examined the first moment of S4-Cy5 binding >1 s for all pre-16S transcripts. The cumulative probability distributions of association times showed that S12 accelerated specific S4-Cy5 binding, whether S12 was present during or after transcription (Figure 5b). This suggests that S12 favors recognition of the 5WJ by S4, and that S12 need not be present with S4 to act.

Because the likelihood of stable S4-Cy5 binding increased dramatically when S12 was present during transcription, we also measured the apparent association time for S4-Cy5 binding >20 s, as above (Figure 5c). The cumulative probability distributions showed that, when S12 is present during transcription, S4-Cy5 binding reached a higher maximum probability compared to when S12 was added after transcription or not at all (Table 2). However, the apparent association rates of long-lived S4-Cy5 binding events, $k_{on,app}$, were similar within error between the different conditions, indicating that the effective on-rate had not changed in the presence of S12 (Figure 5c and Table 2). This is consistent with an RNA chaperone activity of S12, in which S12 accelerates proper folding of the rRNA, thereby generating a larger fraction of rRNAs that are competent to stably interact with S4.

## 4. Discussion

How the rRNA folds during and after transcription is critical for ribosome biogenesis, which is coupled with pre-rRNA synthesis in cells. Estimates of ribosome synthesis kinetics in *E. coli* [30] imply that the primary assembly RP S4 is recruited to the rRNA soon after its binding site is transcribed [31]. In vitro, however, S4 struggles to stably recognize its binding site due to improper folding of the newly made pre-16S rRNA [18]. This dichotomy implies that cells possess mechanisms for facilitating proper rRNA folding during transcription.

Here, we examined how RPs facilitate the binding of protein S4 to the 16S rRNA, which is one of the first complexes formed during 30S ribosome assembly. Surprisingly, we find that RP S12 alone can accelerate stable association of S4 (Figure 1 and Figure 2), although S12 stably joins the complex much later during 30S assembly. Protein S12 only binds weakly with assembly intermediates [32] and pulse-chase mass spectrometry experiments revealed that S12 has a very slow on-rate during reconstitution relative to other RPs [3]. Consistent with these earlier results, we observe that S12 can only interact transiently with the pre-16S rRNA in single-molecule experiments (Figure 3).

There are at least two mechanisms by which S12 may facilitate S4-rRNA complex formation: S12 may co-bind the rRNA with S4 to stabilize the complex, or S12 may independently change the folding path of the S4 binding site, acting as a canonical RNA chaperone. In the mature subunit, S12 binds the 16S 5WJ opposite S4 (Figure S1). Therefore, the two proteins could cooperatively stabilize each other’s interactions with the rRNA when they co-bind the rRNA. However, S12 is unlikely to improve the thermodynamics of S4 binding, as native S4-rRNA complexes are already very stable [13], and S4 binds before S12 in the assembly map. Moreover, it is unlikely that the two proteins occupy the same rRNA under the conditions of our experiments (≤1 per 200 s at 100 nM S12). Instead, our results show that S12 increases the effective rate of stable recruitment, rather than the lifetimes of the S4 complexes (Figure 2 and Figure 5), by acting on the rRNA.

Protein S12 has been previously suggested to act as a general RNA chaperone that facilitates splicing of a group I intron and the hammerhead ribozyme [22]. Interestingly, S12 also binds other RNAs weakly and non-specifically, with only a slight binding preference for unstructured RNA [22]. Despite only interacting with the newly transcribed RNA for short periods of time (~0.5 s), S12 is able to elicit an effect on the rRNA folding path, leading to increased binding of S4. Because we observe that S12 must be present in a high concentration in order to facilitate S4 binding (Figure 3e), it is likely that there is a low probability that S12 successfully chaperones the RNA at each encounter. Similar to other RNA-binding proteins with chaperone activity [33], S12 possesses a broad RNA binding surface, with flexible loops and tails that could help refold the RNA during these short interactions [34]. It is unclear if S12 is acting as a chaperone by interacting with its normal binding site, or if S12 is helping to refold the entire rRNA through nonspecific interactions.

Our order-of-addition experiments suggest that S12 acts on the rRNA independently of S4, since the presence of S12 during transcription followed by its removal leads to even more stable S4 binding post-transcription than when S12 was present together with S4 (Figure 4 and Figure 5). Similarly, S12 does not have to be present during group I intron splicing to improve the splicing efficiency [22]. Moreover, our data suggest that S12 may perform better on rRNAs during transcription than on refolded rRNAs (Figure 5). Altogether, the results support a model in which S12 binds to the rRNA while it is still relatively unstructured to help form native-like initial structures and prevent the rRNA from misfolding. Rapid and transient binding may prevent the accumulation of unproductive S12-rRNA complexes that could inhibit S4 binding.

During assembly in vivo, stable recruitment of S4 must occur soon after transcription of the 5WJ in order to nucleate assembly of the 5′ domain. In our minimal in vitro system, S4 is still unable to bind the rRNA stably during transcription, even in the presence of S12 (Figure 1). Furthermore, S12 only increases the fraction of rRNAs competent for S4 addition to ~20% (Figure 2c). While it is not known how many transcribed rRNAs are ultimately assembled into a mature 30S subunit, it is likely that cells contain redundant mechanisms to accelerate rRNA folding and facilitate binding of S4 and other RPs during transcription. These chaperone mechanisms could include other RPs not tested here, such as RP S1, which has been shown to have RNA chaperone activity [35,36,37,38]. There is evidence that general RNA chaperones function during ribosome assembly, including Hfq, CspA, CsdA, and other RNA binding proteins [33,39]. Future work examining other chaperones will be important in order to characterize the mechanism of the earliest steps in ribosome biogenesis during transcription.

## Figures and Tables

**Table 1:** Lifetimes of S4 complexes. Lifetimes (*τ*) and amplitudes (*a*) of all binding events, from maximum likelihood triple-exponential fits of the unbinned dwell times for S4-Cy5 in the presence of RPs.

RP Added	N_mol_	$\tau_{1}$ (s)	$\tau_{2}$ (s)	$\tau_{3}$ (s) ^2^	$a_{1}$ ^1^	$a_{2}$	$a_{3}$ ^2^
S4 alone	226	0.47 ± 0.01	3.83 ± 1.01	44 ± 11	0.957 ± 0.008	0.037 ± 0.007	0.003 ± 0.008
+100 nM S12	200	0.71 ± 0.02	4.78 ± 0.76	60 ± 13	0.893 ± 0.012	0.089 ± 0.011	0.018 ± 0.012
+20 nM S8	176	0.63 ± 0.02	5.79 ± 1.03	60 ± 21	0.941 ± 0.009	0.053 ± 0.009	0.006 ± 0.009
+100 nM S5	183	0.67 ± 0.01	4.43 ± 0.93	118 ± 29	0.978 ± 0.006	0.020 ± 0.006	0.002 ± 0.006
+50 nM S16	130	0.52 ± 0.02	2.35 ± 0.52	16 ± 3.8	0.923 ± 0.014	0.060 ± 0.015	0.016 ± 0.015

^1^ All distributions are dominated by short events (<1 s). ^2^ The uncertainties in $\tau_{3}$ and a_3_ are large, owing to the few long-lived binding events relative to short events, and $\tau_{3}$ is underestimated because some long-lived events extend beyond the end of the movie.

**Table 2:** Exponential fit parameters for apparent association rates of long-lived S4-rRNA interactions. Cumulative density plots from Figure 5c were fit to a single-exponential rate equation, in which A is the amplitude of plateau and $k_{on,app}$ is the apparent association rate for S4-Cy5 binding events > 20 s.

Experiment	N_mol_	$k_{on,app}$ (s^−1^)	A
S4 alone post-txn	153	0.0043 ± 0.0018	0.11 ± 0.02
S12 added co-txn	143	0.0047 ± 0.0004	0.59 ± 0.02
S12 added post-txn	151	0.0028 ± 0.0012	0.43 ± 0.09

## Data Availability

The data presented in this study are available on request from the corresponding author.

## Supplementary Materials

The following supporting information can be downloaded at: https://www.mdpi.com/article/10.3390/biom13060951/s1, Figure S1: Binding sites of S4 and S12 in *E. coli* 16S rRNA after [40].; Figure S2: Presence of RP S16 or S5 does not influence S4-Cy5 binding dynamics on pre-16S rRNAs.; Figure S3: Maximum likelihood estimation (MLE) analysis of S4-Cy5 dwell times in the presence of different RPs.; Figure S4: Probability density histograms and maximum likelihood estimation (MLE) fits of S4-Cy5 binding to pre-16S rRNAs post-transcription, as outlined in Figure 4; Table S1: MLE analysis of S4-Cy5 binding in the presence and absence of S12 during and after transcription.

## References

[1] Noller H.F. (2005). RNA Structure: Reading the Ribosome. Science.

[2] Stern S., Powers T., Changchien L.-M., Noller H.F. (1989). RNA-Protein Interactions in 30S Ribosomal Subunits: Folding and Function of 16S rRNA. Science.

[3] Talkington M.W.T., Siuzdak G., Williamson J.R. (2005). An assembly landscape for the 30S ribosomal subunit. Nature.

[4] Adilakshmi T., Bellur D.L., Woodson S.A. (2008). Concurrent nucleation of 16S folding and induced fit in 30S ribosome assembly. Nature.

[5] Kim H., Abeysirigunawarden S.C., Chen K., Mayerle M., Ragunathan K., Luthey-Schulten Z., Ha T., Woodson S.A. (2014). Protein-guided RNA dynamics during early ribosome assembly. Nature.

[6] Held W.A., Mizushima S., Nomura M. (1973). Reconstitution of *Escherichia coli* 30 S Ribosomal Subunits from Purified Molecular Components. J. Biol. Chem..

[7] Nowotny V., Nierhaus K.H. (1988). Assembly of the 30S subunit from *Escherichia coli* ribosomes occurs via two assembly domains which are initiated by S4 and S7. Biochemistry.

[8] Ridgeway W.K., Millar D.P., Williamson J.R. (2012). Quantitation of ten 30S ribosomal assembly intermediates using fluorescence triple correlation spectroscopy. Proc. Natl. Acad. Sci. USA.

[9] Davis J.H., Tan Y.Z., Carragher B., Potter C.S., Lyumkis D., Williamson J.R. (2016). Modular Assembly of the Bacterial Large Ribosomal Subunit. Cell.

[10] French S.L., Miller O.L. (1989). Transcription mapping of the *Escherichia coli* chromosome by electron microscopy. J. Bacteriol..

[11] Powers T., Daubresse G., Noller H.F. (1993). Dynamics of In Vitro Assembly of 16 S rRNA into 30 S Ribosomal Subunits. J. Mol. Biol..

[12] Ramaswamy P., Woodson S.A. (2009). Global Stabilization of rRNA Structure by Ribosomal Proteins S4, S17, and S20. J. Mol. Biol..

[13] Gerstner R.B., Pak Y., Draper D.E. (2001). Recognition of 16S rRNA by Ribosomal Protein S4 from *Bacillus stearothermophilus*. Biochemistry.

[14] Sapag A., Vartikar J.V., Draper D.E. (1990). Dissection of the 16S rRNA binding site for ribosomal protein S4. Biochim. Biophys. Acta BBA-Gene Struct. Expr..

[15] Vartikar J.V., Draper D.E. (1989). S4-16 S ribosomal RNA complex: Binding constant measurements and specific recognition of a 460-nucleotide region. J. Mol. Biol..

[16] Powers T., Noller H.F. (1995). A Temperature-dependent Conformational Rearrangement in the Ribosomal Protein S4 16 S rRNA Complex. J. Biol. Chem..

[17] Mayerle M., Bellur D.L., Woodson S.A. (2011). Slow Formation of Stable Complexes during Coincubation of Minimal rRNA and Ribosomal Protein S4. J. Mol. Biol..

[18] Rodgers M.L., Woodson S.A. (2019). Transcription Increases the Cooperativity of Ribonucleoprotein Assembly. Cell.

[19] Bellur D.L., Woodson S.A. (2009). A Minimized rRNA-Binding Site for Ribosomal Protein S4 and Its Implications for 30S Assembly. Proc. Natl. Acad. Sci. USA.

[20] Duss O., Stepanyuk G.A., Puglisi J.D., Williamson J.R. (2019). Transient Protein-RNA Interactions Guide Nascent Ribosomal RNA Folding. Cell.

[21] Williamson J.R. (2000). Induced fit in RNA-protein recognition. Nat. Struct. Mol. Biol..

[22] Coetzee T., Herschlag D., Belfort M. (1994). *Escherichia coli* proteins, including ribosomal protein S12, facilitate in vitro splicing of phage T4 introns by acting as RNA chaperones. Genes Dev..

[23] Culver G.M., Noller H.F. (1999). Efficient reconstitution of functional *Escherichia coli* 30S ribosomal subunits from a complete set of recombinant small subunit ribosomal proteins. RNA.

[24] Culver G.M., Noller H.F. (2000). In vitro reconstitution of 30S ribosomal subunits using complete set of recombinant proteins. Methods Enzym..

[25] Davanloo P., Rosenberg A.H., Dunn J.J., Studier F.W. (1984). Cloning and expression of the gene for bacteriophage T7 RNA polymerase. Proc. Natl. Acad. Sci. USA.

[26] Butler E.T., Chamberlin M.J. (1982). Bacteriophage SP6-specific RNA polymerase. I. Isolation and characterization of the enzyme. J. Biol. Chem..

[27] Hickerson R., Majumdar Z.K., Baucom A., Clegg R.M., Noller H.F. (2005). Measurement of Internal Movements within the 30S Ribosomal Subunit Using Förster Resonance Energy Transfer. J. Mol. Biol..

[28] Hua B., Han K.Y., Zhou R., Kim H., Shi X., Abeysirigunawardena S.C., Jain A., Singh D., Aggarwal V., Woodson S.A. (2014). An improved surface passivation method for single-molecule studies. Nat. Methods.

[29] Friedman L.J., Gelles J. (2015). Multi-wavelength single-molecule fluorescence analysis of transcription mechanisms. Methods.

[30] Lindahl L. (1975). Intermediates and time kinetics of the in vivo assembly of *Escherichia coli* ribosomes. J. Mol. Biol..

[31] Chen S.S., Sperling E., Silverman J.M., Davis J.H., Williamson J.R. (2012). Measuring the dynamics of *E. coli* ribosome biogenesis using pulse-labeling and quantitative mass spectrometry. Mol. Biosyst..

[32] Held W.A., Nomura M. (1973). Rate-determining step in the reconstitution of *Escherichia coli* 30S ribosomal subunits. Biochemistry.

[33] Woodson S.A., Panja S., Santiago-Frangos A. (2018). Proteins That Chaperone RNA Regulation. Microbiol. Spectr..

[34] Tompa P., Csermely P. (2004). The role of structural disorder in the function of RNA and protein chaperones. FASEB J..

[35] Bear D.G., Ng R., Van Derveer D., Johnson N.P., Thomas G., Schleich T., Noller H.F. (1976). Alteration of polynucleotide secondary structure by ribosomal protein S1. Proc. Natl. Acad. Sci. USA.

[36] Kolb A., Hermoso J.M., Thomas J.O., Szer W. (1977). Nucleic acid helix-unwinding properties of ribosomal protein S1 and the role of S1 in mRNA binding to ribosomes. Proc. Natl. Acad. Sci. USA.

[37] Hajnsdorf E., Boni I.V. (2012). Multiple activities of RNA-binding proteins S1 and Hfq. Biochimie.

[38] Lund P.E., Chatterjee S., Daher M., Walter N.G. (2020). Protein unties the pseudoknot: S1-mediated unfolding of RNA higher order structure. Nucleic Acids Res..

[39] Andrade J.M., dos Santos R.F., Chelysheva I., Ignatova Z., Arraiano C.M. (2018). The RNA-binding protein Hfq is important for ribosome biogenesis and affects translation fidelity. EMBO J..

[40] Brodersen D., Clemons W., Carter A., Wimberly B.T., Ramakrishnan V. (2002). Crystal structure of the 30 s ribosomal subunit from Thermus thermophilus: Structure of the proteins and their interactions with 16 s RNA. J. Mol. Biol..

