# Supplementary material for: Ribosomal Protein S12 Hastens Nucleation of Co-Transcriptional Ribosome Assembly
Source: Biomolecules. 2023 Jun 6;13(6):951. doi: 10.3390/biom13060951 (PMC10296100; doi:10.3390/biom13060951)
Supplement: Supplementary file 1 [file biomolecules-13-00951-s001.zip › TableS1 copy.pdf]

Supplemental Table S1. MLE analysis of S4-Cy5 binding in the presence and absence of S12 during and after transcription.

**Experiment**	N_{mol}	τ_1 (s)	τ_2 (s)	τ_3 (s)	a_1	a_2	a_3
S4 alone post-txn	153	0.67 ± 0.01	5.59 ± 1.99	101.86 ± 20.91	0.925 ± 0.007	0.075 ± 0.007	0.000 ± 0.07
S12 added co-txn	143	0.94 ± 0.03	5.65 ± 0.66	42.74 ± 7.54	0.781 ± 0.017	0.182 ± 0.016	0.036 ± 0.017
S12 added post-txn	151	1.06 ± 0.02	7.70 ± 0.71	77.33 ± 17.25	0.881 ± 0.011	0.114 ± 0.010	0.004 ± 0.011
