# Supplementary figures and images for: Ribosomal Protein S12 Hastens Nucleation of Co-Transcriptional Ribosome Assembly
Source: Biomolecules. 2023 Jun 6;13(6):951. doi: 10.3390/biom13060951 (PMC10296100; doi:10.3390/biom13060951)

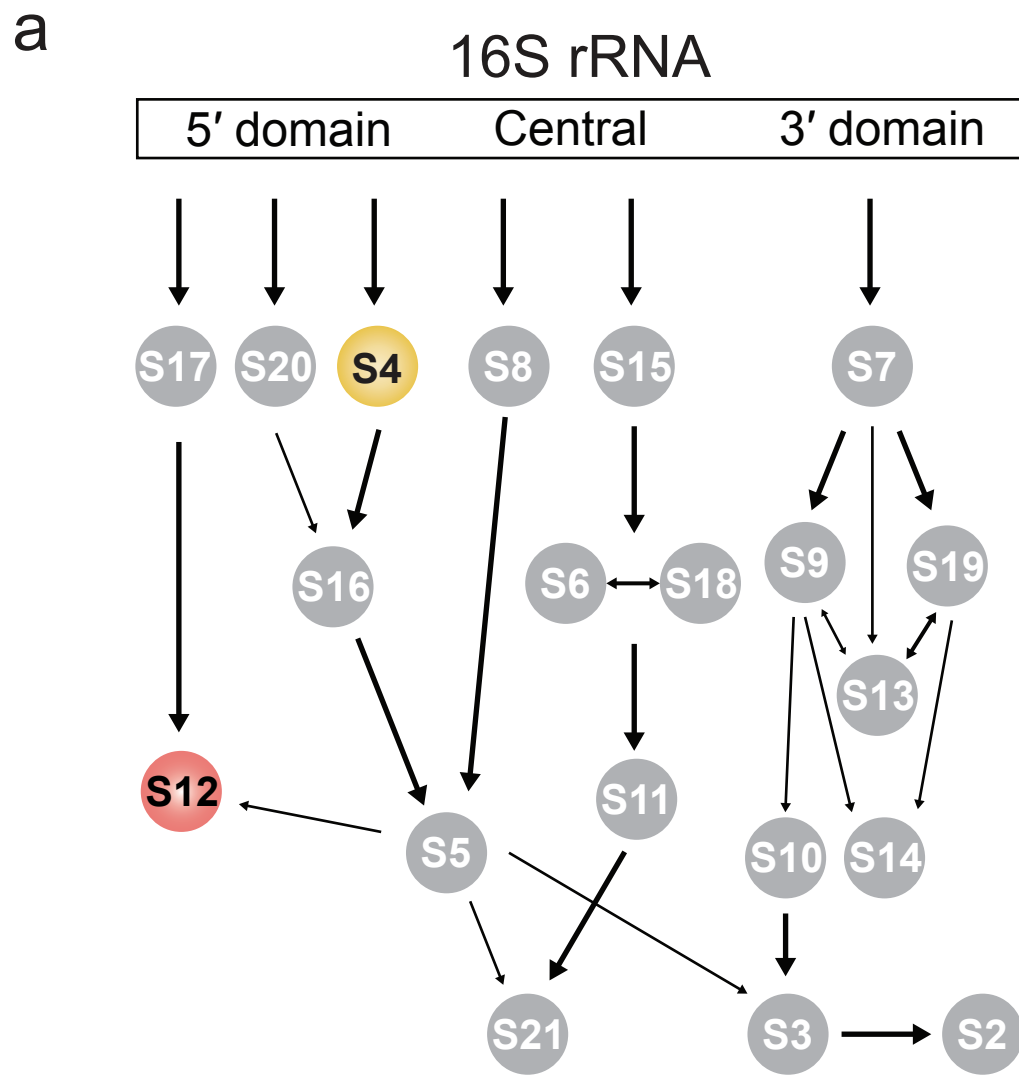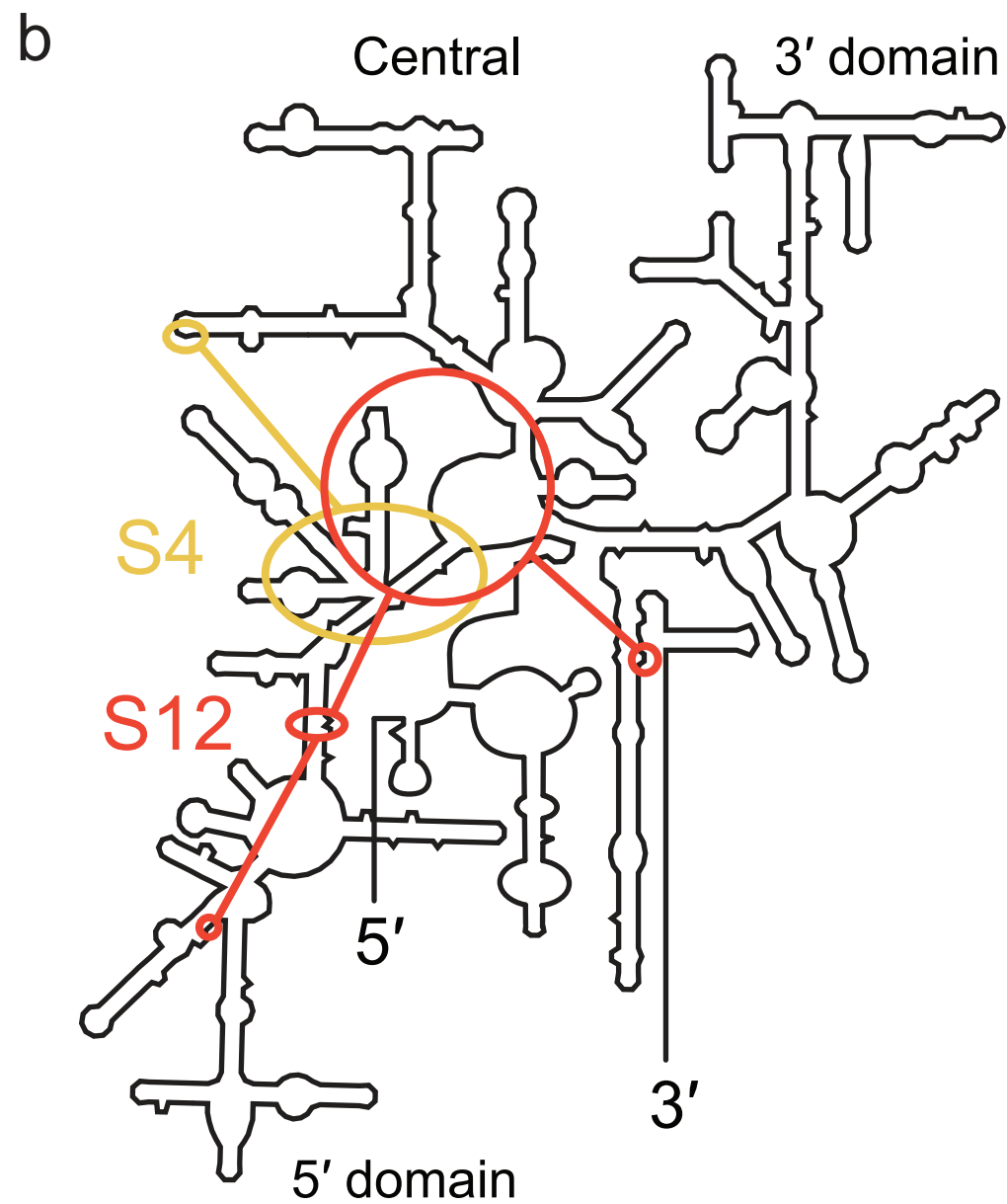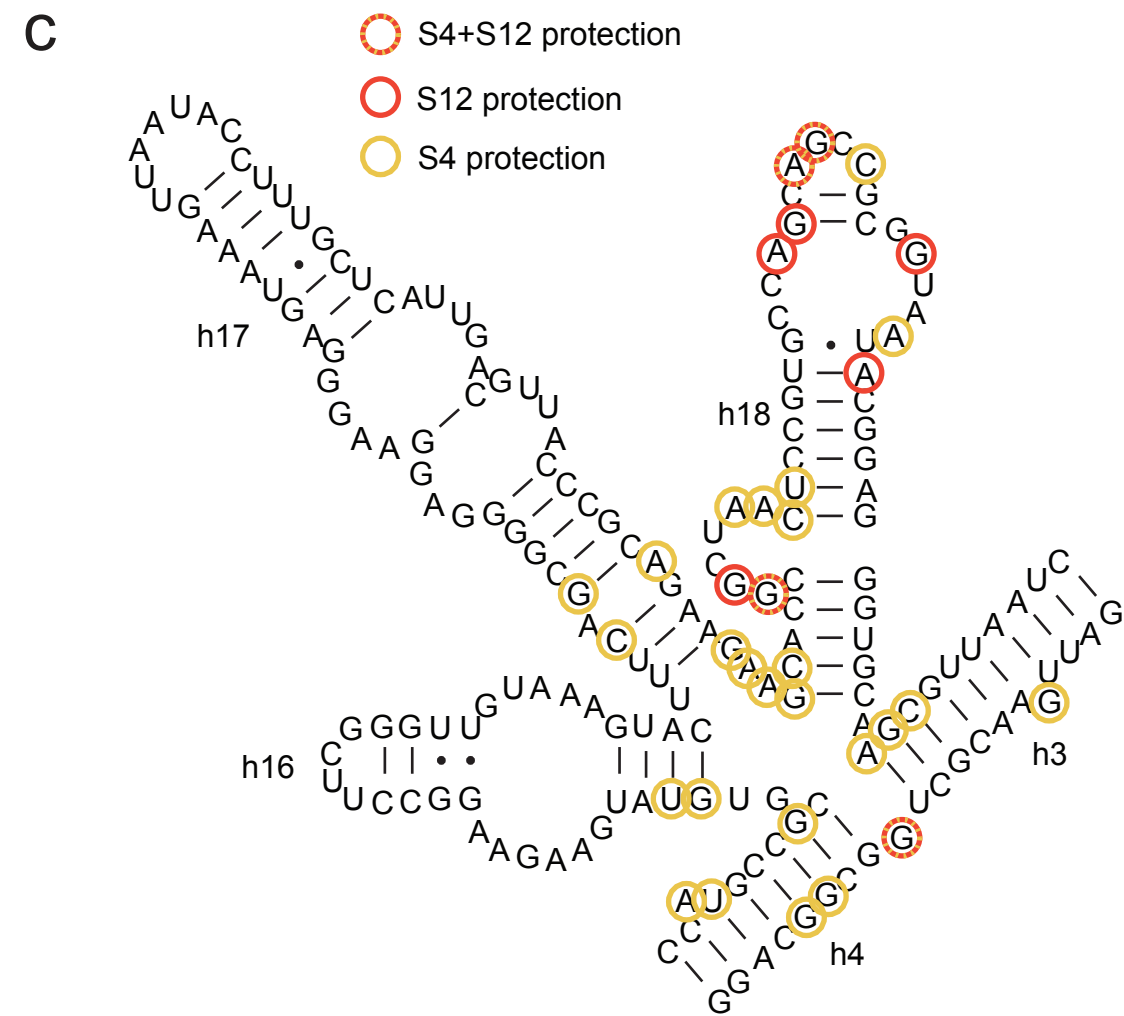

Supplement: Supplementary file 1 [file biomolecules-13-00951-s001.zip › FigureS1.pdf]

a

**S4-Cy5 alone**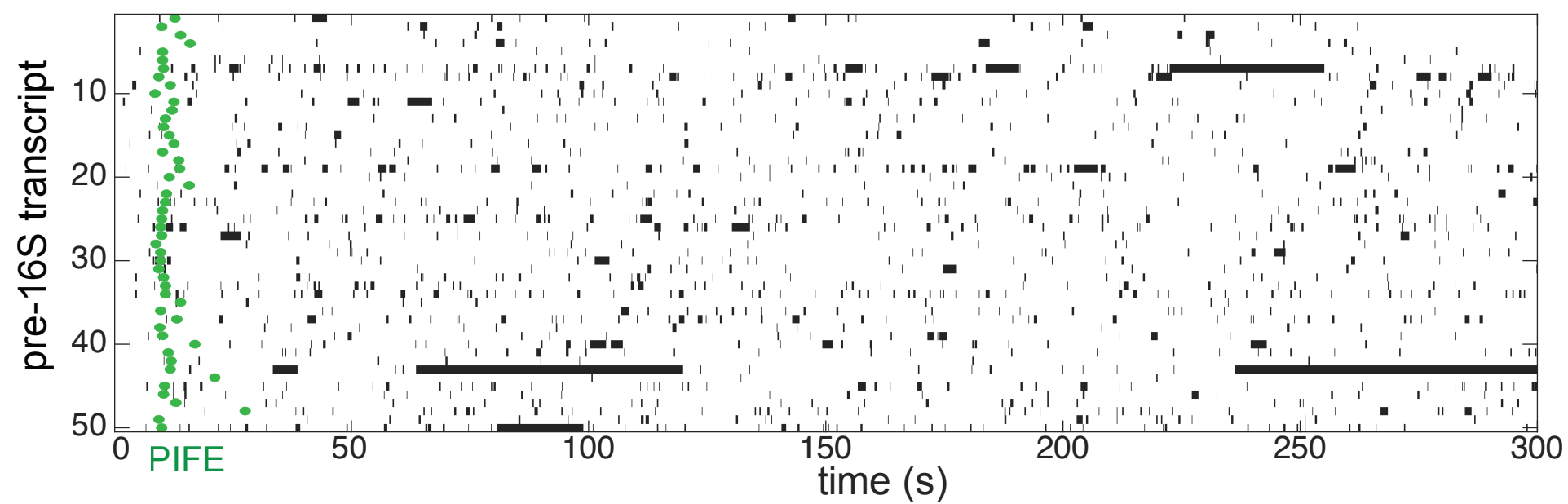

b

**100 nM S5**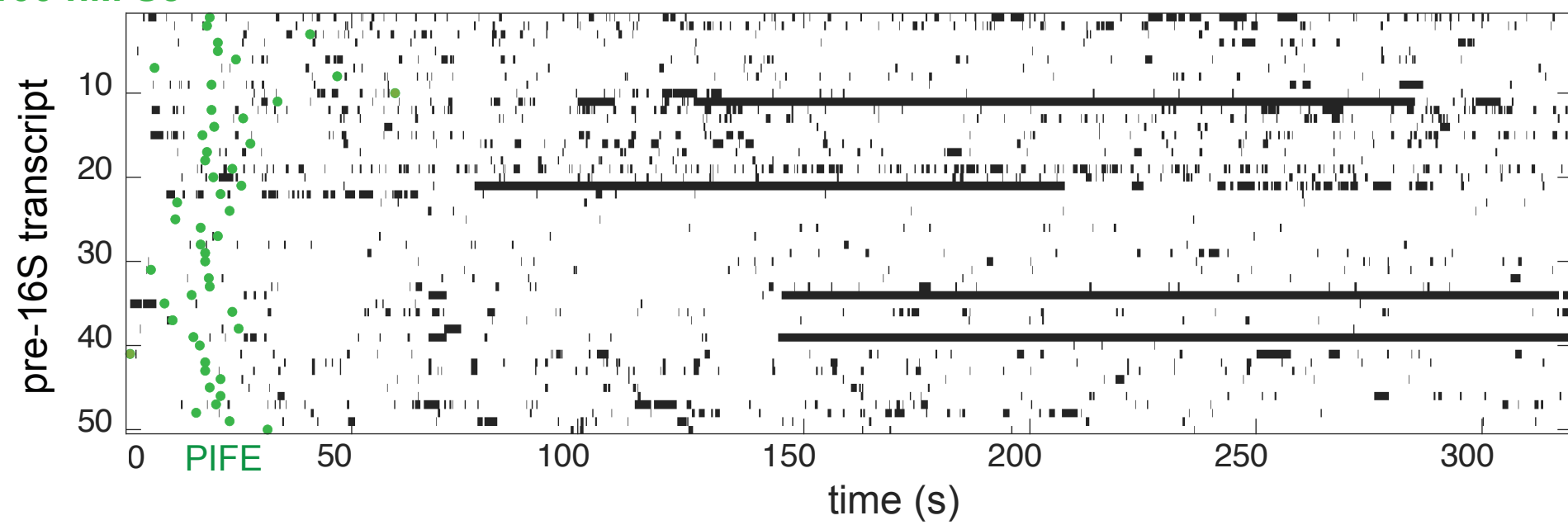

c

**50 nM S16**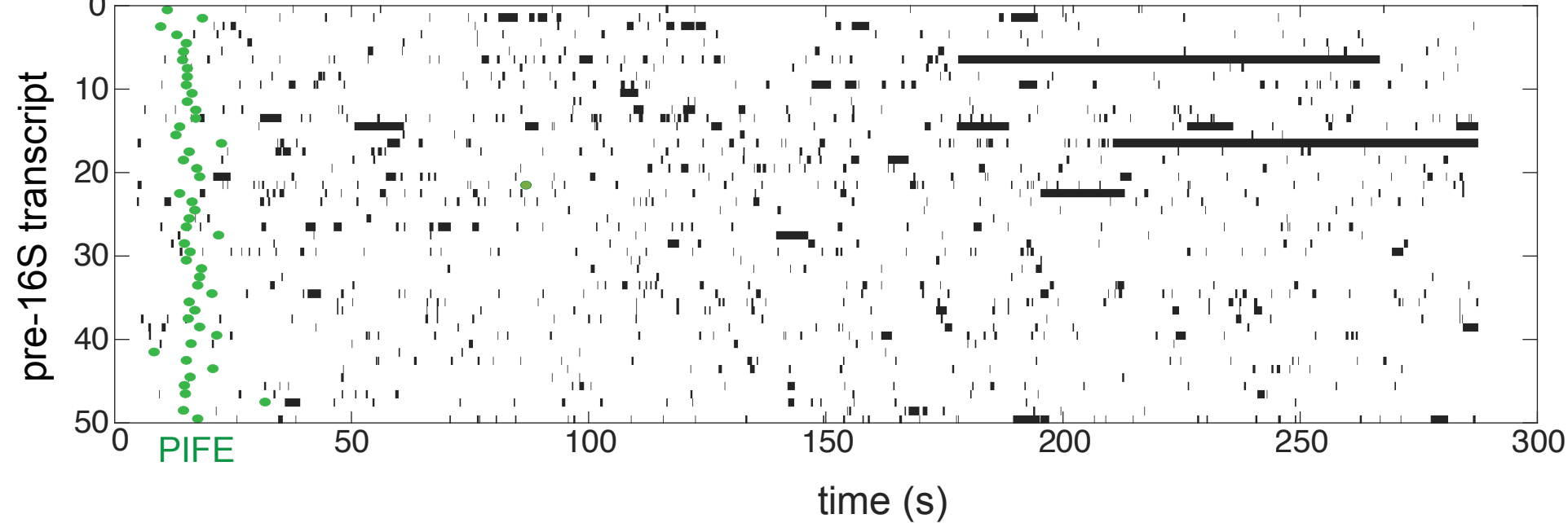

Supplement: Supplementary file 1 [file biomolecules-13-00951-s001.zip › FigureS2.pdf]

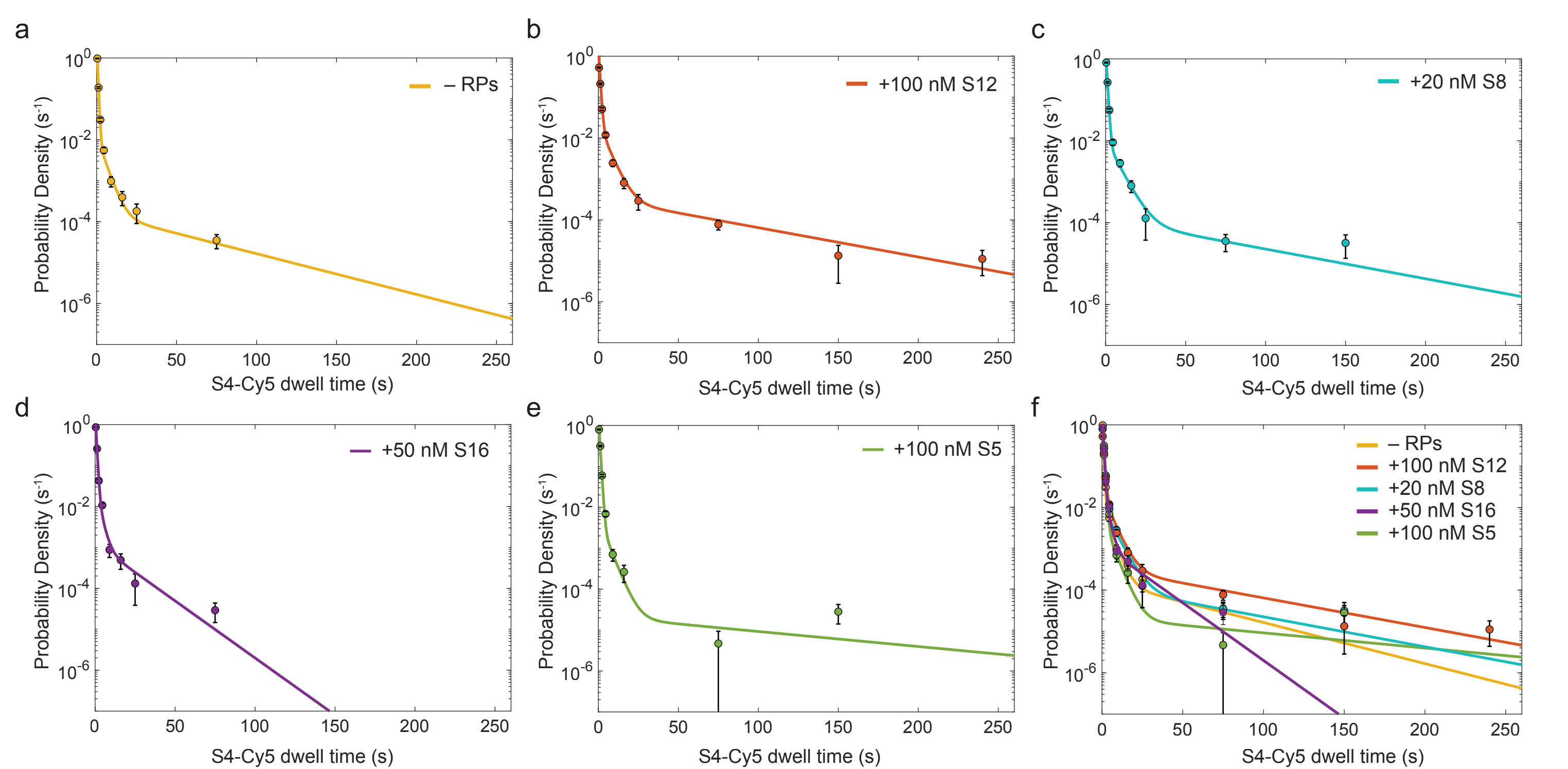

Supplement: Supplementary file 1 [file biomolecules-13-00951-s001.zip › FigureS3.pdf]

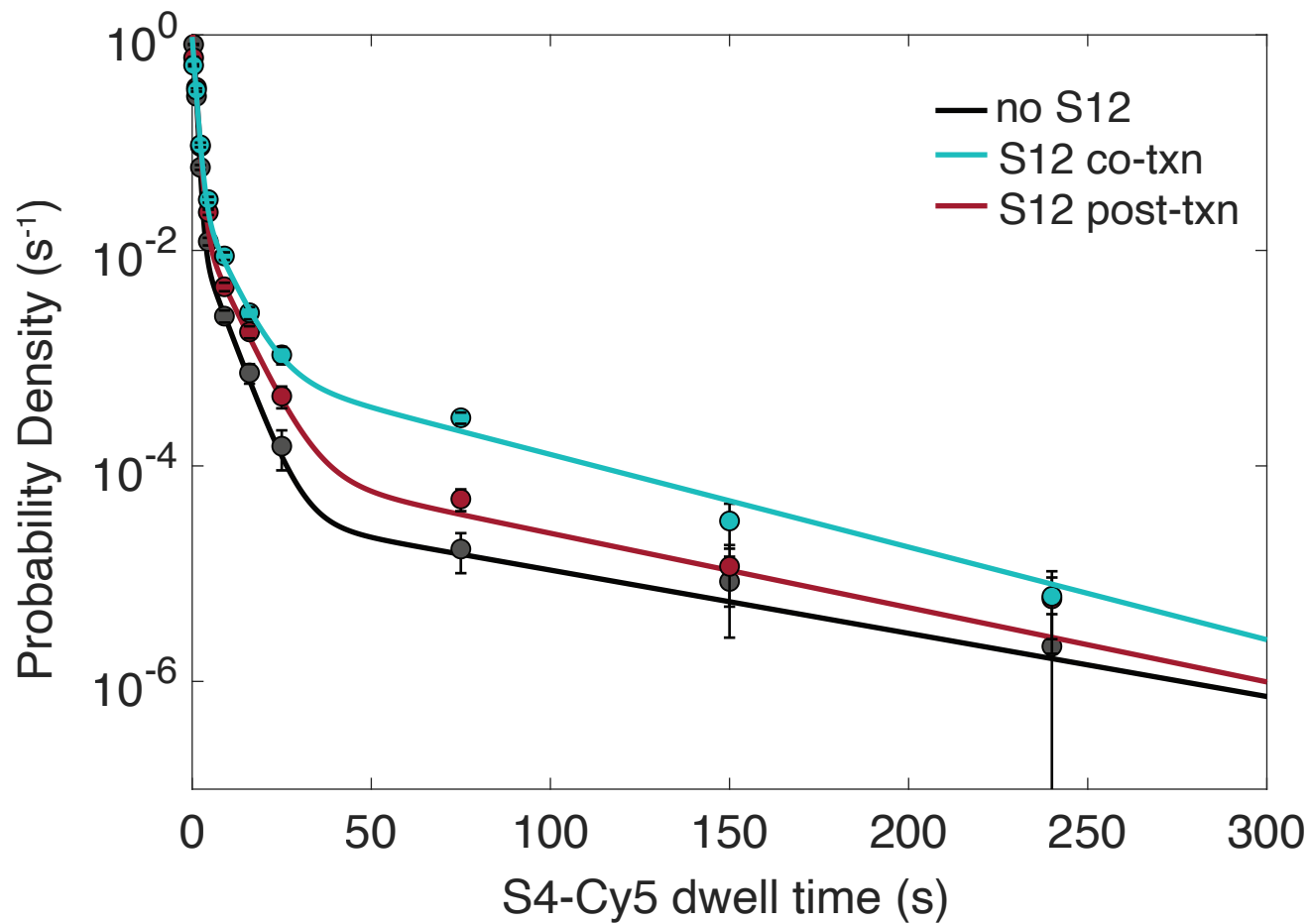

Supplement: Supplementary file 1 [file biomolecules-13-00951-s001.zip › FigureS4.pdf]
